# Supplementary figures and images for: ALDH1A3 Contributes to Radiation-Induced Inhibition of Self-Renewal and Promotes Proliferative Activity of p53-Deficient Glioblastoma Stem Cells at the Onset of Differentiation
Source: Cells. 2024 Oct 31;13(21):1802. doi: 10.3390/cells13211802 (PMC11545341; doi:10.3390/cells13211802)

Figure S1rev2

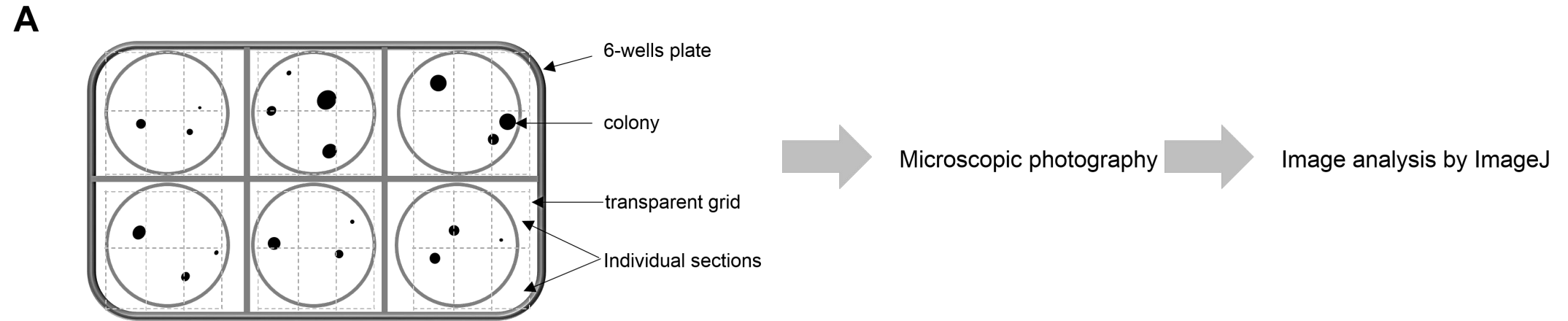

**B**

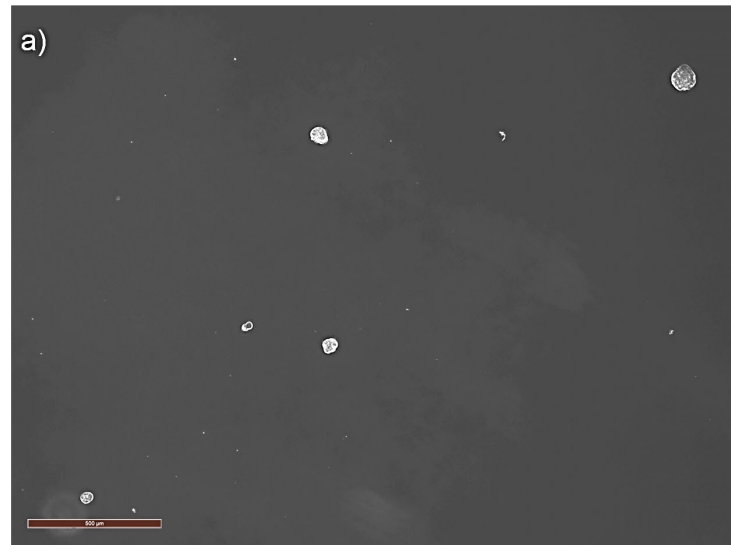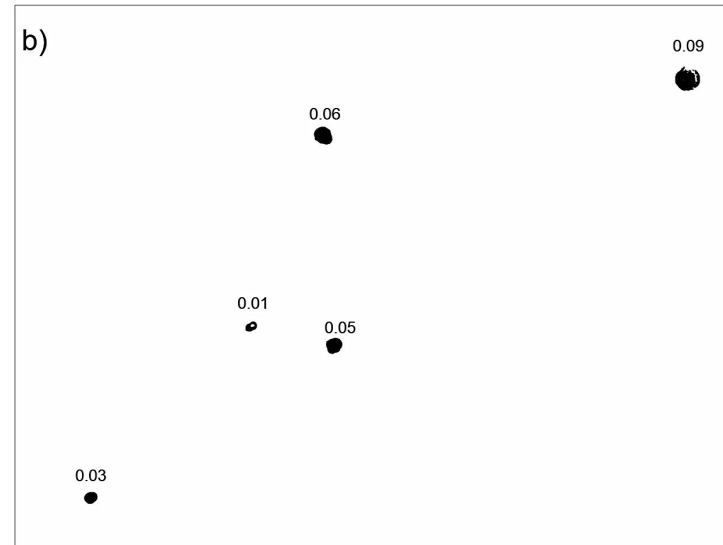

c)

| Object | Area (inch <sup>2</sup> ) |
|--------|---------------------------|
| 1      | 0.05                      |
| 2      | 0.01                      |
| 3      | 0.09                      |
| 4      | 0.03                      |
| 5      | 0.06                      |

Supplement: Supplementary file 1 [file cells-13-01802-s001.zip › cells-3231637-supplementary.pdf]
